# Wound Healing and Omega-6 Fatty Acids: From Inflammation to Repair

**DOI:** 10.1155/2018/2503950

**Published:** 2018-04-12

**Authors:** Jéssica R. Silva, Beatriz Burger, Carolina M. C. Kühl, Thamiris Candreva, Mariah B. P. dos Anjos, Hosana G. Rodrigues

**Affiliations:** Laboratory of Nutrients and Tissue Repair, School of Applied Sciences, University of Campinas, Limeira, SP, Brazil

## Abstract

Wound healing is an evolutionarily conserved process that is essential for species survival. Wound healing involves a series of biochemical and cellular events that are tightly controlled, divided into 3 concomitant and overlapping phases: inflammation, proliferation, and remodelling. Poor wound healing or a chronic wound represents a silent epidemic that affects billions of people worldwide. Considering the involvement of immune cells in its resolution, recent studies are focused on investigating the roles of immune nutrients such as amino acids, minerals, and fatty acids on wound healing. Among the fatty acids, much attention has been given to omega-6 (*ω*-6) fatty acids since they can modulate cell migration and proliferation, phagocytic capacity, and production of inflammatory mediators. The present review summarizes current knowledge about the role of *ω*-6 fatty acids in the wound healing context.

## 1. Wound Healing: A Vital Process

Wound healing occurs after a chemical, physical, or biological insult results in epithelial barrier disruption. This process involves activation of platelets, neutrophils, macrophages, endothelial cells, keratinocytes, and fibroblasts; moreover, the production and release of protein mediators (growth factors and cytokines) released by these cell types and lipid mediators (prostaglandins, leukotrienes, thromboxanes, and lipoxins) are needed to coordinate the tissue repair and to reestablish tissue homeostasis [1, 2].

**Figure 1:**
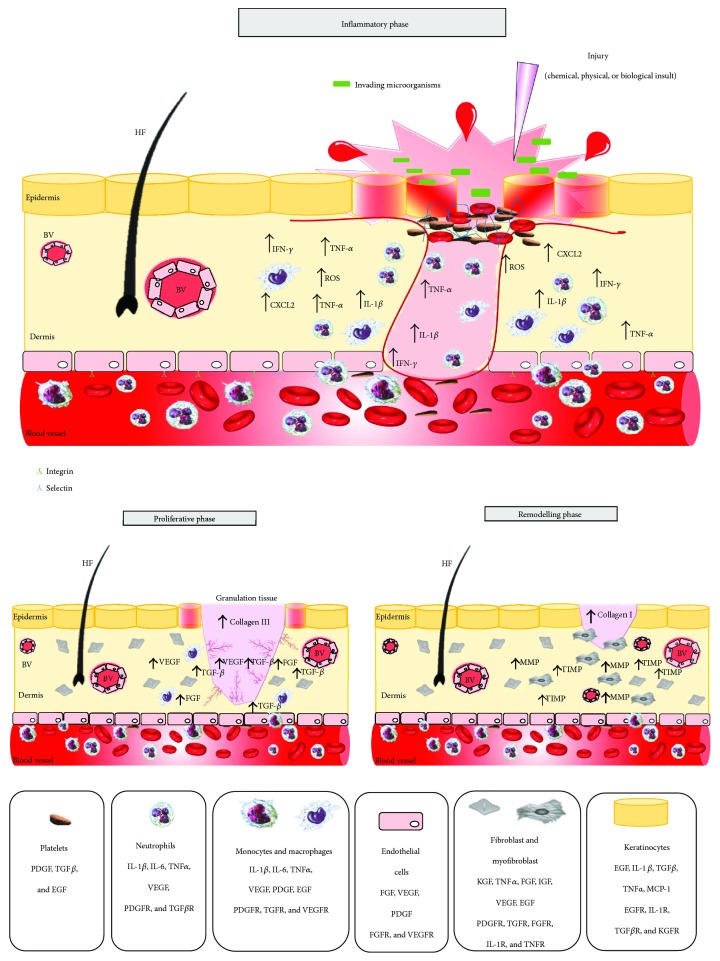
Wound healing process. The illustration shows the inflammatory, proliferative, and remodelling phases of wound healing. Early stages of wound healing include coagulation and activation of inflammatory cells. The proliferative stage involves proliferation of fibroblasts and angiogenesis. The remodelling phase includes restoration of the barrier and contraction of the wound by myofibroblasts. The process is orchestrated by immune cells and growth factors and cytokines and chemokines (listed below) [8]. HF = hair follicle; BV = blood vessels; TNF = tumor necrosis factor; IL-1beta = interleucina 1beta; IL-6 = interleucina 6; ROS = reactive oxygen species; CXCL2 = chemokine (C-X-C motif) ligand 2; IFN-gamma = interferon-gamma; VEGF = vascular endothelial growth factor; TGF-beta = transforming growth factor beta; FGF = fibroblast growth factor; KGF = keratinocyte growth factor; MCP1 = monocyte chemoattractant protein-1; IGF = insulin growth factor; TIMPs = tissue inhibitors of metalloproteinases; MMPs = matrix matalloproteinases; PDGF = platelet-derived growth factor; EGF = epithelial growth factor.

The process is divided into 3 concomitant and overlapping phases: inflammation, proliferation, and remodelling (Figure 1).

After a tissue lesion, the disruption in vasculature blocks the oxygen and nutrient supply to the injured area, leading to a hypoxic condition that induces the production of reactive oxygen species (ROS) and reactive nitrogen species (RNS) [3–5], initiating a coagulation cascade. Blood elements such as platelets, erythrocytes, and fibrin form a framework for the recruitment of immune cells [6, 7]. Platelets produce platelet-derived growth factor (PDGF), transforming growth factor-*β* (TGF-*β*), and epidermal growth factor (EGF) that induce migration and activation of immune cells [8].

The extracellular matrix (ECM), which is composed of fibronectin, fibrinogen, fibrin, thrombospondin, and vitronectin, fills the tissue defect and enables migration of different cell types required for the healing process [9].

Inflammation is the body's natural and essential defence mechanism responsible for combating antigens, restoring homeostasis, and repairing tissue damage [10, 11]. The inflammatory response consists of a variety of events triggered by immune cells, which involves influx of leukocytes to the injured area and production of pro- and anti-inflammatory mediators [12].

Neutrophils are the predominant cells in the first hours after the tissue injury, and they respond to proinflammatory cytokines, such as interleukin-1*β* (IL-1*β*), tumor necrosis factor alpha (TNF-*α*), and interferon gamma (IFN-*γ*) at the lesion site, phagocytizing microorganisms and cellular debris [10, 13]. For microorganism destruction, the degranulation process occurs, releasing granule enzymes such as defensins, cathelicidins, elastase, myeloperoxidase (MPO), lactoferrins, and cathepsins inside the phagosome. In addition to their microbicidal activities, these molecules also act in the chemoattraction of macrophages to the lesion site. They also amplify the production of cytokines and chemokines, such as chemokine C-X-C motif ligand-2 (CXCL2) that will attract macrophages to the wound area. Neutrophils produce IL-1*β*, TNF-*α*, and vascular endothelial growth factor (VEGF) and express PDGF and TGF-*β* receptors [8]. These cytokines also induce the expression of adhesion molecules on the endothelial cell surface that will interact with selectins and integrins expressed in macrophages, facilitating the rolling, attachment, and transmigration of these cells to injured areas [13–15].

Macrophages are phagocytes that have PDGF, TGF-*β*, and VEGF receptors, and thus, they migrate in response to mediators produced by platelets and neutrophils in the injured tissue [8].

After 72 hours, macrophages are the predominant cells at the wound site, and they release growth factors (VEGF, PDGF, and EGF) and cytokines (IL-1*β*, IL-6, and TNF-*α*) that promote the migration of other cells such as fibroblasts and endothelial cells [10, 16]. They also produce prostaglandins that activate endothelial cells and act as potent vasodilators, affecting the permeability of microblood vessels [17].

The lack of control in the amplitude and time to resolve inflammation is one of the factors that most influence the genesis of chronic inflammatory diseases, such as cardiovascular diseases, diabetes, cancer, asthma, dementia, and Alzheimer's disease [11, 12, 18], as well as chronic wounds.

The proliferative stage begins within the first 48 hours and can unfold up to the 14th day after a tissue injury [17]. This phase is characterized by angiogenesis and fibroplasia, restoring the blood vessels and forming the granulation tissue [10].

Angiogenesis is the formation of new blood vessels from preexisting vessels and it is initiated by growth factors such as VEGF, PDGF, and fibroblast growth factor (FGF) [10, 19]. Fibroplasia is the formation of granulation tissue, and its main characteristic is proliferation of fibroblasts in response to PDGF, TGF-*β*, FGF, IL-1, and TNF*α*. At this time, the production of collagen occurs, and there is a release of growth factors such as keratinocyte growth factor (KGF), TNF-*α*, FGF, insulin growth factor (IGF), VEGF, and EGF [8, 16]. Then, the provisional matrix initially formed is replaced by granulation tissue composed of fibroblasts, granulocytes, macrophages, and blood vessels in complex with collagen bundles that form the basis for cell adhesion and migration, growth, and differentiation [10, 19].

The remodelling phase occurs approximately from the 21st day after injury and can last for years. During this period, there is intense production and digestion of collagen as well as the substitution of collagen III for collagen I. These events are aimed at maintaining the fibres in the same direction as in unwounded tissue to reestablish its functions and mechanical forces [20].

Matrix metalloproteinases (MMPs) are zinc-dependent endopeptidases that degrade ECM components (collagens), and their gene expression is regulated at the transcriptional level by cytokines and growth factors as well as by their natural inhibitors, tissue inhibitors of metalloproteinases (TIMPs) [21].

In all of the processes cited above, it is important to emphasize that exogenous and endogenous factors can modulate such events and influence the healing process. More specifically, systemic disorders, such as diabetes, immunosuppression, and venous stasis as well as those resulting from external agents, such as the use of corticotherapy and smoking, can hinder the early closure of the wound.

Chronic wounds are defined as wounds with a full thickness in depth and a slow healing tendency. The time an open wound needs to remain to define chronicity is still not well established, ranging from 4 weeks to more than 3 months [22]. Including diabetic foot ulcers, venous leg ulcers, and pressure ulcers, they represent a silent epidemic that affects a large fraction of the world population, becoming a public health problem [23, 24].

Complications of chronic wounds include infection that can lead to lower extremity amputations and impacts on health and life quality of patients because they cause pain and suffering, loss of function, loss of productivity, depression, distress and anxiety, social isolation, prolonged hospital stays, and chronic morbidity or even death [25].

Moreover, the treatment of chronic wounds causes economic expenditures by the individual and the healthcare system, and therefore, they are a matter of political interest [26]. In the USA, for example, chronic wounds affect 6.5 million patients, with nearly US$ 50 billion spent on treatment of chronic wounds and complications related to them per year [22, 25].

In developed countries, 1 to 2% of the population will experience a chronic wound throughout their lives [22] due to population ageing and increases in noncommunicable diseases such as obesity, diabetes, and cancer [27, 28].

Animal and human studies have shown that in the elderly population (age over 60 years), there is an increase in the number of cases of poor wound healing and chronic wounds due to delayed T-cell infiltration, decreased chemokine production, and reduced macrophage phagocytic capacity, in addition to delayed reepithelization, collagen synthesis, and angiogenesis [28].

Alterations of the peripheral nervous system with decreased protective sensation and foot deformities enhance the risk of chronic skin ulcerations on the lower extremities of diabetic subjects, mainly on the foot, that affect 15% of diabetic patients [29], and five-year postamputation, the mortality rate is 50–59% [30].

Chronic inflammation, insufficient angiogenic response, production of ROS greater than antioxidant capacity, collagen accumulation, dysfunction of migration and proliferation of fibroblast and keratinocytes, and an imbalance between the accumulation and degradation of ECM are some of the mechanisms responsible for poor healing in diabetic patients [1, 28].

These scenarios show the necessity for studies that investigate possible therapies that accelerate tissue repair, reducing the susceptibility to infections [31].

## 2. Omega-6 Fatty Acids: From Inflammation to Regeneration

For decades, nutritional supplementation was mainly used to avoid nutritional deficiency. However, currently it is being recognized that adequate levels of essential nutrients can prevent as well as treat some disturbances [32].

Fatty acids are carboxylic acids formed by hydrogen and carbon atoms [33]. Based on the presence of double bonds, fatty acids are classified as saturated (no double bonds) or unsaturated (with double bonds). Among the unsaturated fatty acids, there is also a classification that takes into account the number of unsaturations: monounsaturated fatty acids (MUFAs) present with one double bond on acyls (the main food source is olive oil) and polyunsaturated fatty acids (PUFAs) contain two or more double bonds [33].

PUFAs are classified by the position of the first double bound counting from the methyl terminal. Then, when the first double bond is at the 6th carbon atom from the methyl terminal, PUFAs are called omega-6, *ω*-6, or n-6. They include linoleic acid (LA, C18 : 2 *ω*-6), an essential fatty acid because it cannot be synthesized by the human body. LA can be “stretched” and desaturated into other *ω*-6 fatty acids, such as *γ*-linolenic (GLA, 18 : 3 *ω*-6) and the arachidonic acid (AA, 20 : 4 *ω*-6). Moreover, biohydration of linoleic acid by bacteria (anaerobic bacteria such as *Butyrivibrio fibrisolvens*) in the gut of animals and the action of Δ9 desaturation of 18 : 1 *trans*-11 in animal tissue can generate conjugated linoleic acid (CLA) [34].

Fatty acids alter skin structural and immunological status since they constitute the stratum corneum, and they can alter the permeability of the skin. They also interfere with maturation and differentiation of the stratum corneum and inhibit production of proinflammatory eicosanoids, reactive species (ROS and RNS), and cytokines, thus influencing the inflammatory response and possibly wound healing [35–38].

The objective of this study was to review the scientific literature on the relationship between omega-6 fatty acids (linoleic, conjugated linolenic, gamma-linolenic, and arachidonic acid) and the wound healing process.

### 2.1. Linoleic Acid

Linoleic acid (LA, 18 : 2 *ω*-6, *cis*-9, 12-octadecadienoic acid) is the PUFA most commonly consumed in the human diet, being mainly found in safflower, corn, and sunflower oils, present in medium quantities in soy, sesame, and almond oils, and in smaller quantities in canola, olive, coconut, and palm oils [39].

#### 2.1.1. Effects of LA on Tissue Repair

In developing countries, creams with linoleic acid are used to treat wounds. One of the first studies in this area described that topical application of a solution containing 1.6 g of essential fatty acid (mainly linoleic acid) prevented pressure ulcers in hospitalized patients [40]. This improvement was related to better hydration and elasticity. In this study, the control group received a solution with 1.6 g of mineral oil, which is a liquid paraffin. Although frequently used in baby creams to maintain hydration, in the present study, only 22% of the patients in the control group presented a hydrated skin. On the other hand, 98% of the patients treated with a linoleic solution showed a hydrated skin. These results indicate that maintenance of hydration is a mechanism by which linoleic acid improves wound healing.

In 2008, Magalhães et al. [41] did not observe any effects of topical application of medium chain triglycerides (caprylic, capric, caproic, and lauric acids), linoleic acid, soy lecithin, or a vitamin A and E mixture on wound healing in rats. The main problem with this protocol is the composition of the mixture tested and the control used, since the control group received a 0.9% NaCl solution without antioxidant vitamins or fatty acids.

Other studies described positive effects of oils rich in linoleic acid such as lucuma nut oil (LNO) and pumpkin oil on wound healing [42, 43]. The major constituents of LNO are LA (38.9%), oleic acid (27.9%), and palmitic and stearic acids (18.6 and 8.9%, resp.). Rojo et al. [42] used two different models to prove the beneficial effects of LNO: the zebrafish (*Danio rerio*) model and a CD-1 murine model. At first, LNO (20–100 *μ*g/mL) was added to a zebrafish larva plate after a tail primordial cut. Through fluorescence microscopy and image analysis of fluorescent endothelial cells from zebrafish, it was observed that LNO accelerated the regeneration. This prohealing effect was attributed to increases in angiogenesis. However, the authors evaluated the number of fluorescent endothelial cells and not the number of new vessels formed. Angiogenesis is a complex process that involves release of proangiogenic factors, such as VEGF, PDGF, and FGF, as well as proteolytic degradation of basement membrane, and the migration, proliferation, and organization of endothelial cells in a tube form [44]. Angiogenesis is a crucial step for proper wound healing since it reestablishes the oxygen and nutrient supply.

LNO was also tested in a CD-1 murine model. For this, a wound was induced in the back region of mice and topically treated with 200, 500, or 100 *μ*g of LNO daily. Corroborating the results with zebrafish, LNO (500 and 1000 *μ*g) also induced a more rapid wound closure in CD-1 mice. The authors hypothesize that this effect could be related to the anti-inflammatory actions of the fatty acids present in the LNO [42]; however, they did not show any results that could support this hypothesis.

Linoleic acid has been thought to be behind the effects of pumpkin oil on wound healing. Pumpkin oil is constituted, mainly by LA (50%), oleic acid (25%), and palmitic acid (15%) [43]. This high content of LA was correlated with improvement in wound closure, since it shortened bleeding time, suggesting a stabilization of fibrin and consequent migration of fibroblast; it augmented hydroxyproline content, possibly due to fibroblast activation; and it reduced the number of infiltrating macrophages in wound tissue 11 days after lesion induction. Altogether, these results indicate that topical treatment with pumpkin oil accelerates tissue repair, mainly due to the effects of LA [43].

Considering these results with LA-rich oils, some groups have tested the effects of pure LA on wound healing. The use of isolated fatty acids ensures that the observed effects are not due to minor oil compounds or a combination of fatty acids.

In this context, it was also reported that there were beneficial effects of pure LA topically applied into wounds. BALB/c mice treated with pure LA (30 *μ*M) for 20 days exhibited accelerated tissue repair 48 hours after wound induction [45]. This result was related to increased production of nitric oxide (NO). NO is a free radical derived from L-arginine oxidation through the nitric oxide synthase (NOS) activity. After an inflammatory insult, inducible nitric oxide synthase (iNOS) is expressed in immune cells and produces a large amount of NO that will generate other free radicals, expanding the inflammatory response [46]. NO plays important roles such as activation of macrophages and fibroblasts, induction of collagen synthesis, and the proliferation of keratinocytes during wound healing, thus accelerating reepithelization [47].

However, in Wistar rats, topical treatment with pure LA did not alter the wound area, although there was an increase in wet wound weight (oedema) and in neutrophil numbers, indicating a positive effect on the migratory response during the inflammatory phase [48].

Another approach used to investigate LA effects on wound healing is oral supplementation.

Wistar rats orally supplemented with pure LA (0.22 g per kg body weight) by gavage during the 5 days prior to wound induction had an increased inflammatory response 1 hour (initial stage of inflammation) after skin injury. This proinflammatory effect was characterized by an increase in inflammatory cell influx into the wound site due to elevations in hydrogen peroxide (H_2_O_2_) production and chemokine release. On the other hand, 24 hours later, LA reduced the activation of nuclear transcription factor (NF-*κ*B) and then diminished the production of proinflammatory cytokines such as IL-1*β* and IL-6. At the same time, there was an elevation in AP-1 (activator protein-1) activation. AP-1 is a transcription factor that induces the expression of genes related to proliferation of keratinocytes and fibroblasts, which are two important cells involved in the proliferative phase of wound healing. Therefore, LA accelerated the inflammatory phase of wound healing, allowing the next phase (proliferation) to start early and accelerating wound healing over a period of 7 days [49].

More recently, the same protocol was tested in diabetic Wistar rats; the results showed that LA positively modulates tissue repair not only by accelerating the inflammatory phase but also by inducing angiogenesis. During the proliferative phase (7 days), it was observed that LA increased the number of vessels in the wound tissue, which was related to an elevation in VEGF concentration and ANGPT-2 (angiopoietin-2) expression [38]. VEGF and ANGPT-2 are proangiogenic factors essential for new vessel formation. VEGF induces ANGPT-2 expression, which primes endothelial cells to respond to inflammatory cytokines, thereby augmenting the migration and proliferation of endothelial cells [50].

Taken together, these studies demonstrate that linoleic acid can improve wound healing due to its mechanical properties and by modulating the cellular response, increasing the migration and functions of inflammatory and endothelial cells as well as inducing angiogenesis at the wound site.

#### 2.1.2. Mechanisms of Action of LA

The mechanisms described so far to explain the effects of LA on wound healing involve inflammatory responses of neutrophils and macrophages.

Neutrophils are the first cell type recruited to the inflammatory site, being determinants for the healing process [51]. To analyse the effects of LA on neutrophil migration, an air pouch was induced into the dorsal region of Wistar rats treated with LA (100 *μ*M), and 4 hours later, the exudate was collected and the cells were counted. LA increased neutrophil influx to the pouches [48], corroborating the results described in wound tissue. This effect on migration can be explained by the induction of adhesion molecules such as L-selectin on neutrophil surfaces [52]. Neutrophil recruitment is a highly regulated process that involves at least four steps: rolling, activation, adhesion, and transmigration. Through the intravital microscopy assay, it was observed that LA also elevated leukocyte-endothelium interactions (rolling and adhesion) [52].

Once in the injured site, neutrophils produce cytokines, chemokines, ROS, and other molecules to expand the inflammatory response. Measuring intra or extracellular ROS production, Hatanaka et al. [53] demonstrated that LA increased anion superoxide and H_2_O_2_ in a dose-dependent manner. The authors tested 5 different techniques (luminol- and lucigenin-amplified chemiluminescence, cytochrome c, hydroethidine, and phenol red reduction) and described that LA interfered with luminol and cytochrome c reactions, jeopardizing the ROS results [53]. In the wound healing context, ROS production is the first event that occurs after tissue disruption due to hypoxia [54]. Low concentrations of H_2_O_2_ are important to support wound healing [55] since ROS not only disinfects the injured area but also acts as signalling messengers regulating gene expression [56] and cellular function such as migration [57] and cytokine production [58].

Inflammation control is crucial to tissue repair, since chronic inflammation can worsen the wound. In this sense, LA has also shown a beneficial effect since it increases the release of proinflammatory mediators in the initial inflammation phase (1–4 hours) and reduces them in the resolution phase (18–48 hours) [52].

Another important cell type that is involved in inflammatory responses is the macrophage. As observed with neutrophils, LA reduced the production of IL-1*β*, IL-6, and VEGF in the absence of LPS, although it accelerated IL-1*β* release and decreased IL-10 synthesis when cells were stimulated with LPS. However, LA did not affect ROS production (superoxide anion, hydrogen peroxide, and NO) as well as the lipid mediators, prostaglandin E_2_ (PGE2), leukotriene B_4_ (LTB_4_), and 15(S)-hydroxyeicosatetraenoic acid (15[2]-HETE) [59]. Lipid mediators are a class of inflammatory molecules derived from the metabolization of arachidonic [60], eicosapentaenoic (EPA), or docosahexaenoic (DHA) acids. Classes 2 and 4 are derived from AA and exhibit more proinflammatory effects, increasing migration, production of cytokines, and ROS. On the other hand, classes 3 and 5 are derived from EPA and DHA and are related to anti-inflammatory effects. More recently, a new class of lipid mediators derived from omega-3 fatty acids (EPA and DHA) were described, the maresins, resolvins, and protectins that exert proresolution effects, resolving inflammation [61]. During the inflammatory response, it is important that there is a shift between proinflammatory molecules to proresolution to limit the damage induced by exacerbated inflammation.

During the proliferation and remodelling phases, fibroblasts, endothelial cells, and keratinocytes play important roles in producing growth factors that orchestrate the reconstruction of vessels and induce wound contraction [62]. In this context, Rojo et al. [42] described a promigratory effect of LNO (60 *μ*g/mL) on human fibroblasts, which was related to an increase in vinculin expression. Vinculin is a focal adhesion protein essential for fibroblasts-ECM interactions [63] involved in wound contraction.

One important aspect not fully clarified is if LA must be metabolized to exert its effects on cellular functions or if it acts as an effector molecule. To answer this question, some studies have described G-protein coupled receptors (GPCR or GPR) as responsible for fatty acid effects [64, 65]. GPR is a class of seven transmembrane receptors involved in a broad spectrum of cellular responses [64]. Among GPR, GPR40 has been described as a sensor for LA, oleic acid (OA), CLA, and other long chain fatty acids [65, 66]. In HaCaT cells (keratinocyte cell line), once activated, it reduced the production of cytokines (CCL-5 and CCL17) and suppressed allergic inflammation in skin [67], and then, it could be involved in the effects of LA on wound healing. These results indicate that LA can modulate immune response by acting as an effector molecule. However, considering the importance of LA to cellular membranes, it is possible that the results observed are due to its metabolization as well. More studies are needed to clarify this point.

In conclusion, it has been shown that LA-rich oil or pure LA modulates cellular functions such as migration, production of ROS, cytokines, and chemokines, expression of adhesion molecules, and interaction with ECM. These alterations seem to be related to improvements in tissue repair.

### 2.2. Conjugated Linoleic Acid (CLA)

The presence of conjugated linoleic acid (CLA) was first reported in 1930 [68], but only in the 1980s was CLA described as a bioactive dietary constituent, and the interest in CLA's effects has increased due its anticarcinogenic properties and reduction of adipose tissue mass observed in mice [69].

CLA comprises a mix of positional and geometric isomers of linoleic acid with a single pair of conjugated double bonds. CLA is formed during LA biohydration by bacteria in the gut of ruminant animals, and thus, the main natural sources of CLA are ruminant meats (beef and lamb) and dairy products (milk and cheese) [69, 70].

At least 28 CLA isomers are known, but the *cis*-9, *trans*-11 (c9, t11) is the most abundant form of CLA in nature, and nutritional supplements are a mixture of c9, t11 and *trans*-10, *cis*-12 (t10, c12) CLA [71, 72]. Initially, it was thought that the effects of CLA were global, and the results were due to interactions between its two main isomers: c9, t11 and t10, c12. However, later evidence suggested that the physiological effects of CLA may be different between the isomers, animal species (rats and mice), and cell types [73].

The last decade has seen a plethora of claims, supported by animal and cell lineage models, that dietary CLA intake is associated with potential health benefits [70]. These include reduction in fat deposition, protection from atherosclerosis and cancer, and enhanced immunity [69, 74].

Although preclinical data suggest benefits of CLA supplementation, clinical findings in humans have yet to show evidence of a positive effect, and even the findings in animals are still controversial [73].

Some studies revealed that CLA can induce adverse effects such as fatty liver, insulin resistance, and lipodystrophy [75]. Thus, it is recommended that ingestion of a balanced diet with natural sources of CLA be followed.

#### 2.2.1. Effects of CLA on Tissue Repair

Mice fed a diet supplemented with 0.5% or 1% CLA (38% c9, t11 CLA; 39% t10, c12 CLA; 3% c9, c11 CLA; and 1% t9, t11 CLA) for 2 weeks presented a reduction in wound area (1% CLA) that was related to an increase in antioxidant defences [76]. ROS are essential to protect the organism against invading bacteria and other microorganisms; moreover, they are important to intracellular signalling. However, excessive production of ROS or impaired detoxification of these molecules causes oxidative stress [54]. To understand the prohealing effect, the authors measured malondialdehyde (MDA) content in the liver, a marker of lipid peroxidation, and the expression of antioxidant enzymes at the wound site. Mice supplemented with CLA had a reduced MDA content and increased CuZn superoxide dismutase (SOD) and MnSOD protein expression, showing an antioxidant effect of this fatty acid, which can explain its benefit on wound healing. At the same time, they described a reduction of phosphorylated inhibitor kappa B alpha (pI*κ*B*α*) protein expression at the end of the inflammatory phase of wound healing [76]. In the cytoplasm, NF-*κ*B is found complexed with I*κ*B. Once phosphorylated, I*κ*B releases NF-*κ*B that translocates to the nucleus and induces the expression of genes related to inflammatory responses [77]. Therefore, the reduction in pI*κ*B*α* indicates that NF-*κ*B is in the cytoplasm, and the expression of proinflammatory genes is reduced. To show this, the expression of cyclooxygenase-2 (COX-2) and HO-1 was evaluated. CLA reduced the protein expression of these inflammatory genes, confirming its inhibitory effect on NF-*κ*B activation [76].

In the carcinogenic context, topical application of CLA to hairless mouse skin also reduced COX-2 expression due to inhibition of NF-*κ*B activation in the skin [78]. To elucidate the CLA effects on the NF-*κ*B pathway, it was described that this fatty acid downregulated the catalytic activity of I*κ*B kinase (IKK*α*/*β*), mitogen-activated protein kinase (p38 MAPK), and protein kinase B (Akt) [78]. We suggest Zhang et al. [77] for a comprehensive review of the NF-*κ*B signalling pathway.

#### 2.2.2. Mechanisms of Action of CLA

The mechanisms by which CLA modulates immune function are not completely elucidated, but they include regulation of prostaglandin and cytokine production, since it has been observed that CLA reduces COX-2 expression and modulates NF-*κ*B activation [76, 78, 79].

Peripheral blood mononuclear cells (PBMC) treated with t10, c12 CLA (100 *μ*M) for 24 hours diminished TNF-*α* production. This effect seems to be isomer-specific since treatment with c9, t11 CLA (100 *μ*M) or LA (100 *μ*M) had no effect on TNF-*α* concentration [80].

Cho et al. [81] suggested that t10, c12 CLA has a priming effect on polymorphonuclear (PMN) and mononuclear cells isolated from dogs. PMN or mononuclear cells directly treated with CLA did not alter TNF-*α* production. Thus, they took this preconditioned medium and added it to a new cell culture. This preconditioned medium increased TNF-*α* concentrations and augmented the oxidative burst activity and phagocytic capacity of PMN and mononuclear cells [81]. When the recombinant anti-TNF-*α* antibody was added to this preconditioned medium, the effects were abolished, suggesting that the effects of CLA are mediated by TNF-*α* released from PBMC.

Taken together, these results showed that dietary administration of CLA can improve wound healing due to antioxidant and anti-inflammatory effects in the later inflammatory phase of tissue repair.

### 2.3. Gamma Linolenic Acid (GLA)

Gamma-linolenic acid (GLA, 18 : 3 *ω*-6) is an omega-6 fatty acid formed through LA metabolization, due to delta-6-desaturase action [82]. It is found in plant seed oils, such as borage, black current seed, and primrose oil [83]. The most common form of GLA consumption is through oral supplementation with GLA-rich oil capsules, mainly from evening primrose oil (EPO) [84].

GLA has been investigated in chronic inflammatory diseases such as rheumatoid arthritis [83, 85, 86], atopic dermatitis, acne vulgaris, and psoriasis [87–89] due to its anti-inflammatory effects. GLA can be converted into dihomo-*γ*-linoleic acid (DGLA), which is metabolized into prostaglandin E1 (PGE1) or 15-hydroxyeicosatrienoic acid (15-HETrE) [82, 89]. These eicosanoids have anti-inflammatory and immunoregulatory effects [85].

#### 2.3.1. Effects of GLA on Tissue Repair

GLA ingestion was also used to treat patients with acne vulgaris [88]. In this study, 45 patients received 2 capsules of borage oil (400 mg of GLA) for 10 weeks, and acne lesion number and severity were assessed as well as inflammation by histological analysis. The GLA group had a reduction in the lesion number and severity, which could be associated with a reduction in inflammation and interleukin-8 (IL-8) staining demonstrated by histologic analysis [88]. Although the authors speculate that two mechanisms (modulation of inflammation and improvement of skin quality) could explain their results, no other analyses were made of their samples. Therefore, it is not possible to affirm how GLA had beneficial effects on acnes vulgaris.

Ingestion of GLA-rich oil capsules was also related to clinical improvement of atopic dermatitis (AD) [89]. The clinical effect was positively correlated with plasma GLA and DGLA concentrations after 4 weeks of capsule consumption.

#### 2.3.2. Mechanisms of Action of GLA

Considering the relevance of macrophages in inflammatory processes such as arthritis and wound healing, it is of great value to investigate the effects of GLA on their functions.

In the RAW 264.7 macrophage cell line, GLA concentrations (100 to 200 *μ*M) reduced the expression of inducible nitric oxide synthase (iNOS) and consequently the NO concentration [90]. GLA also inhibited the expression of COX-2 and prointerleukin-1, suggesting a reduction in inflammatory responses. To explain these results, the authors evaluated the expression of proteins involved in the NF-*κ*B pathway. GLA diminished I*κ*B phosphorylation and degradation, blocking the transmigration of NF-*κ*B to the nucleus, which was confirmed by the reduction in nuclear p65 protein expression. Altogether, these results explain the reduced activation of NF-*κ*B in GLA-treated macrophages [90].

More studies are necessary to prove the beneficial effects of GLA on wound healing.

### 2.4. Arachidonic Acid

Arachidonic acid (AA, 20 : 4 *ω*-6) is the second most abundant fatty acid in injured tissue after a tissue lesion [91]. Once released from membrane phospholipids by phospholipase A_2_, AA is metabolized by cyclooxygenases and lipoxygenases and produces the eicosanoids [92].

Eicosanoids are a wide variety of 20-carbon bioactive lipid products that include prostaglandins (PGs) and thromboxanes (TXs) of series 2 and leukotrienes (LTs) of series 4, lipoxins (LXs), hydroxyeicosatetraenoic acids (HETEs), and epoxyeicosatrienoic acids (EETs) [93] that modulate inflammatory responses. They are highly potent, short-lived molecules that act locally and have been strongly associated with a variety of physiological and pathological processes including cancer, inflammatory diseases, and wound healing [92]. In the wound healing process, the effects of AA are associated mainly with the production of eicosanoids, because they are abundant in the wound bed [94].

The AA metabolites are predominantly proinflammatory because they stimulate the chemotaxis of inflammatory cells, increase the activity of elastase that degrades extracellular proteins, and impair the formation and remodelling of healing tissue [94].

#### 2.4.1. Effects of AA on Tissue Repair

Considering that AA generates eicosanoids and that these molecules modulate tissue repair, an AA-enriched diet was tested in an intestinal ischaemia-injured model [95]. The diets were enriched with 0.5 or 5% of AA and administered over 10 days to pigs. After this period, blockage of the mesenteric blood vessels induced an ischaemic ileum injury, and the protective and reparative effects of AA administration were analysed. It was observed there was a protective effect of AA (5%) since the percentage of denuded villus area was reduced in relationship to the control. At the same time, the AA group presented an improvement in recovery since these animals showed a reduction in mucosal-to-serosal flux of ^3^H-mannitol and ^14^C-inulin when compared with the control group (0% of AA), suggesting that the epithelial barrier is more preserved in the AA group [95]. Although AA-enriched diet (5%) does not alter COX-2 mRNA expression, it was observed that there was an increase in PGE_2_ concentration after ischaemic injury [95]. This effect was abolished when animals received indomethacin, a nonselective COX inhibitor. PGE_2_ has been described to be a protective factor that stimulates the recovery of gut injury [96]. One of the mechanisms involved is the induction of angiogenesis due to an increase in VEGF content [97].

In the dextran sodium sulphate-induced inflammatory bowel disease (IBD) model, oral administration of AA (240 mg/kg of body weight) for 8 weeks aggravated inflammation since it increased COX-2, LTB_4_, and TXB_2_ concentrations in colonic tissue. AA also elevated myeloperoxidase (MPO) activity and macrophage infiltration, which reinforces its proinflammatory effect [98].

Epoxyeicosatrienoic acids (EETs) are metabolites produced from AA due to cytochrome P450 (CYP450) activity, predominantly in the endothelium. EETs can stimulate angiogenesis and organ or tissue regeneration [21, 99]. Local application of 11,12- or 14,15-EETs (10 *μ*M/methylcellulose discs) accelerated wound healing due to the increase in MMP2 and MMP7 and reduction in TIMP-1 and TNF-*α* during the proliferative phase of wound healing (12 days). These results indicate that EETs favoured extracellular matrix degradation and endothelial cell migration, two important steps in the angiogenesis process [21]. These positive effects were also confirmed in transgenic animals that exhibit high or low EET. In this model, the wound healing was accelerated in high EET animals due to vascularization [100].

To better explain the roles of lipid mediators during impaired wound healing, a lipidomic approach was carried out in transgenic animals (LIGHT^−/−^) that exhibited an exacerbated inflammatory response characterized by high levels of oxidative stress and cytokines and imbalance between production and degradation of ECM [99]. These animals had increased concentrations of 11-, 12-, and 15-hydroxyeicosatetraenoic acid, leukotrienes (LTD_4_ and LTE), prostaglandins (PGE_2_ and PGF_2_), thromboxane (TXA_2_/TXB_2_), and prostacyclins in early stages (1 day) of wound healing. These results were associated with enhanced coagulation and infiltration of neutrophils in LIGHT^−/−^ when compared with wild type mice [99].

The factors that lead to colitis, ischemia-reperfusion damage, and skin wound are physiologically different as well as the responses observed in these conditions. Although a general inflammatory response is usually observed, which is characterized by recruitment and activation of inflammatory cells, production of cytokines and growth factors as well as the repair of the damaged tissue, there are specificities inherent to each tissue that can change the effect of determined fatty acid.

#### 2.4.2. Mechanisms of Action of AA

In agreement with the *in vivo* studies, AA induced endothelial cell adhesion *in vitro*. Once again, this modulatory effect presented a biphasic pattern as also observed with other fatty acids, such as LA. In the first phase, the positive effect on cell spreading was independent of AA concentration. However, in the later phase, there was an inverse correlation between AA concentration and spreading [101]. Low AA concentrations (20 *μ*M) increased cell spreading, and high AA concentrations (80 *μ*M) reduced it. This effect could be associated with the metabolites generated from AA oxidation. At a low concentration, AA is totally metabolized, and the products can induce cell adhesion. On the other hand, at high concentrations, the reaction is saturated in AA, and the enzymes involved are not sufficient to metabolize all AA available. Then, there is a reduction in AA metabolites and consequently a reduction in cell spreading [101]. AA also randomizes the migration of endothelial cells. This action is related to the loss of direction during migration due to the presence of AA and seems to be inversely correlated with AA concentration [101].

Some of these AA effects, observed in endothelial cells, were also described in human umbilical cord blood-derived mesenchymal stem cells (hUCB-MSCs), and once again, AA concentration was inversely related with the migratory response. The mechanism behind this effect seems to involve GPR40 [91], a membrane receptor for fatty acids [102].

In a very detailed study, Oh et al. [91] demonstrated that AA binds to GPR40 and then induces mammalian target of rapamycin complex 1 (mTORC2) phosphorylation (mTOR^ser2481^) that activates Akt^ser407^, which phosphorylates protein kinase C*ζ* (PKC*ζ*). pPKC*ζ* activates p38, through Sp1 phosphorylation, and increases the expression of matrix metalloproteinases (MMPs). MMP degrades fibronectin, an extracellular matrix component, promoting the migration of hUCB-MSCs.

Altogether, the studies demonstrate that AA and its metabolites promote wound healing due to induction of cell migration and angiogenesis. However, these positive effects are closely related with the concentrations used.

## 3. Summary

Wound healing is an evolutionarily conserved process essential for species' survival. An investigation of factors that improve wound healing is of crucial interest. Experimental and clinical studies indicate that LA improves wound healing due to its biphasic effects on the inflammatory phase of tissue repair (Table 1). CLA seems to have antioxidant and anti-inflammatory effects on the later inflammatory phase of tissue repair, favouring the beginning of the proliferative phase (Table 2). Although less studied, GLA presented positive effects controlling inflammation (Table 3). Studies investigating the effects of AA demonstrated that AA and its metabolites promoted wound healing due to induction of cell migration and angiogenesis (Table 4).

**Figure 2:**
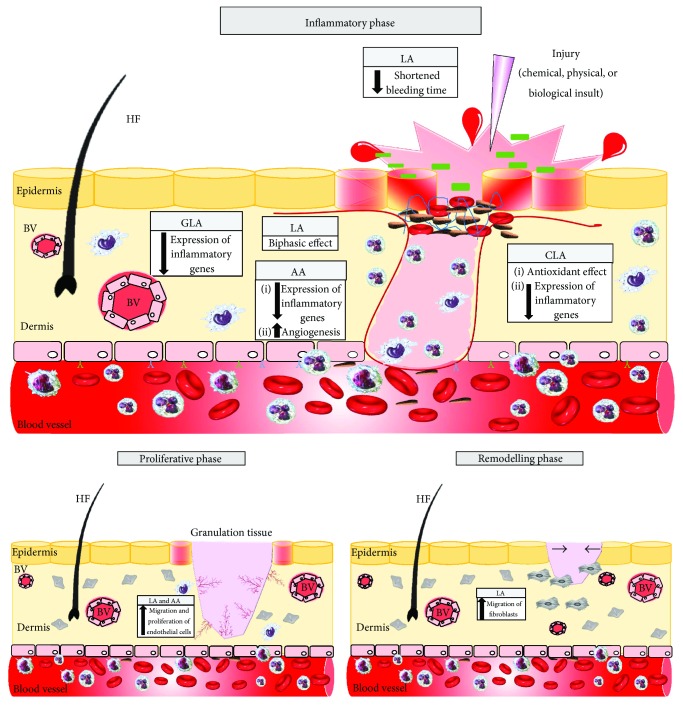
Effects of linoleic acid (LA), conjugated linoleic acid (CLA), gamma linolenic acid (GLA), and arachidonic acid on wound healing phases.

In general, omega-6 fatty acids positively modulate all phases of wound healing, but more studies are necessary to clarify the mechanisms involved (Figure 2).

Clinical studies are essential to establish the strategies of fatty acid administration (topically or orally), the optimal concentrations, and their safety.

## Figures and Tables

**Table 1:** Effects of linoleic (LA) fatty acid.

Fatty acid	Condition	Study model	Treatment time	Dose/concentration	Molecules associated	Effect in tissue repair	Reference
LA	Wound healing	Diabetic Wistar rats	18 days	0.22 g/Kg bw (oral administration)	Increased VEGF and ANGPT-2	Accelerated the inflammatory phase and angiogenesis	[38]
	Pressure ulcers	Healthy humans	21 days	1.6 g EFA with LA extracted from sunflower oil (topical application)	NA	Increased hydration and elasticity.	[40]
	Wound healing	Healthy rats	12 days	0.14 g solution with TGs, LA, vitamins A and E, and soy lecithin (topical application)	NA	No effects	[41]
	Wound healing	Zebrafish	48 hours	10–100 *μ*g/mL of lucuma nut oil	NA	Improved regeneration (100 *μ*g/mL)	[42]
		CD-1 mice	11 days	200, 500, or 1000 *μ*g of lucuma nut oil (topical application)	NA	Improved wound healing and formation of new blood vessels (500 and 1000 *μ*g)	
	Wound healing	Healthy rats	11 days	0.52 *μ*L/mm^2^ of pumpkin oil (topical application)	Increased hydroxyproline content	Accelerated wound closure and bleeding time, improved fibrin stabilization, increased migration of fibroblasts, and reduced infiltration of macrophages	[43]
	Wound healing	Healthy BALB/c mice	20 days	30 *μ*M of pure LA (topical application)	Increased NO production	Accelerated tissue repair	[45]
	Wound healing	Healthy Wistar rats	24 hours	300 *μ*L of pure LA (topical application)	Increased total protein and DNA contents and elevated VEGF-*α* and IL-1	No effect on wound area	[48]
	Wound healing	Healthy Wistar rats	5 days	0.22 g/Kg bw of pure LA (oral administration)	Increased H_2_O_2_ and AP-1 and reduced NF-*κ*B, IL-1*β*, and IL-6	Accelerated the inflammatory phase	[49]
	Neutrophil functions	Intraperitoneal neutrophils from healthy Wistar rats	10 days	0.11, 0.22, and 0.44 g/kg of bw (oral administration)	Increased L-selectin, IL-1*β*, and CINC-2*αβ*	Increased leukocyte-endothelium interactions	[52]
	Neutrophil functions	Intraperitoneal neutrophils from healthy Wistar rats	20 minutes	0, 10, 25, 50, 100, and 200 *μ*M *(in vitro)*	Increased O_2_^−^ and H_2_O_2_ (50 *μ*M)	Increased ROS	[53]
	Macrophage functions	Macrophages from healthy Wistar rats	10 days	0.22 g/Kg bw (oral administration)	Reduced IL-6, VEGF, and IL-10	Modulated cytokine production by macrophages	[59]

Essential fatty acids (EFA); triglycerides (TGs); nitric oxide (NO); Deoxyribonucleic acid (DNA); vascular endothelial growth factor (VEGF); interleukin-1*β* (IL-1*β*); body weight (bw); hydrogen peroxide (H_2_O_2_); activator protein-1 (AP-1); nuclear transcription factor (NF-*κ*B); interleukin-6 (IL-6); angiopoietin-2 (ANGPT-2); cytokine-induced neutrophil chemoattractant-2 (CINC-2*αβ*); reactive oxygen species (ROS); lipopolysaccharides (LPS); interleukin-10 (IL-10); not analysed (NA).

**Table 2:** Effects of conjugated linoleic acid (CLA).

Fatty acid	Condition	Study model	Treatment time	Dose/concentration	Molecules associated	Effect in tissue repair	Reference
CLA	Wound healing	Healthy mice	2 weeks	0.5 or 1% of CLA (diet)	Increased CuZnSOD, and MnSOD and reduced pIkB*α*, COX-2, HO-1, and MDA	Increased the antioxidant defences and reduced the wound area (1%)	[76]
	Hairless skin	Mice	6 hours	0.25 or 1 mg (topical application)	Reduced NF-*κ*B, COX-2, IKK*α*/*β*, MAPK, and Akt	Antitumor (1 mg)	[78]
	Inflammatory diseases	Bovine PBMC	24 hours	100 *μ*M *(in vitro)*	Decreased TNF-*α*	Additional studies are needed	[80]
	Inflammatory diseases	Blood phagocytes isolated from dogs	24 hours	10 *μ*M *(in vitro)*	Increased TNF-*α*	Increased oxidative burst activity and phagocytic capacity	[81]

CuZn superoxide dismutase (CuZnSOD); Mn superoxide dismutase (MnSOD); cicloxigenase-2 (COX-2); malondialdehyde (MDA); nuclear transcription factor (NF-*κ*B); I*κ*B kinase (IKK*α*/*β*); mitogen-activated protein kinase (MAPK); protein kinase B (Akt); tumor necrosis factor *α* (TNF-*α*); peripheral blood mononuclear cells (PBMC); not analysed (NA).

**Table 3:** Effects of gamma linolenic (GLA) fatty acid.

Fatty acid	Condition	Study model	Treatment time	Dose/concentration	Molecules associated	Effect in tissue repair	Reference
GLA	Acne vulgaris	Healthy humans	10 weeks	400 mg (oral administration)	Reduced IL-8	Reduced lesion number, severity, and inflammation	[88]
	Atopic dermatitis (AD)	Humans	12 weeks	320 or 480 mg (oral administration)	NA	Improvement of clinical signs of AD	[89]
	Macrophage functions	RAW 264.7 macrophages		100 to 200 *μ*M *(in vitro)*	Reduced iNOS, NO, COX-2, pro-IL-1, pI*κ*B, and NF-*κ*B	Decreased inflammation	[90]

Interleukin-8 (IL-8); inducible nitric oxide synthase (iNOS); oxide nitric (NO); cicloxigenase-2 (COX-2); prointerleukin-1 (pro-IL-1); nuclear transcription factor (NF-*κ*B); not analysed (NA)

**Table 4:** Effects of arachidonic (AA) fatty acid.

Fatty acid	Condition	Study model	Treatment time	Dose/concentration	Molecules associated	Effect in tissue repair	Reference
AA	Wound healing	hUCB-MSC	24 hours	5 or 10 *μ*M *(in vitro)*	Increased mTOR^ser2481^, Akt^ser407^, PKC*ζ*, and MMPs	Increased cell migration and angiogenesis (10 *μ*M)	[91]
	Intestinal ischemic injury	Pigs	10 days	0.5 or 5% of AA (diet)	Increased PGE_2_	Preservation of epithelial barrier (5%)	[95]
	IBD	Rats	8 weeks	0, 5, 35, or 240 mg/Kg of bw (oral administration)	Increased COX-2, LTB_4_, TXB_2_, and MPO	Increased inflammation and macrophage infiltration	[98]
	Angiogenesis	Porcine endothelial cells	24 hours	0, 20, 50, 60, and 80 *μ*M *(in vitro)*	NA	Increased cell spreading (20 *μ*M) and reduced cell spreading (80 *μ*M)	[101]

Prostaglandin E2 (PGE_2_); inflammatory bowel disease (IBD); body weight (bw); cicloxigenase-2 (COX-2); leukotriene B_4_ (LTB_4_); thromboxane (TXB_2_); myeloperoxidase (MPO); human umbilical cord blood-derived mesenchymal stem cell (hUCB-MSC); mammalian target of rapamycin complex 1 phosphorylation (mTOR^ser2481^); protein kinase B (Akt^ser407^); phosphorylates protein kinase C*ζ* (PKC*ζ*); matrix metalloproteinases (MMPs); not analysed (NA).
